# Neuron-specific enolase and brain-derived neurotrophic factor as developmental neurovascular markers in chronic kidney disease and kidney transplantation

**DOI:** 10.1007/s00467-026-07437-w

**Published:** 2026-07-01

**Authors:** Leah Hernandez, Camillo Tancredi Strizzi, Ylva Tranæus Lindblad, Peter Stenvinkel, Peter Barany, Milan Chromek, Karolina Kublickiene

**Affiliations:** 1 Division of Renal Medicine, Clinical Science, Intervention and Technology (CLINTEC), Karolinska Institutet, 17177 Stockholm, Sweden; 2 Department of Translational Medicine and Surgery, Università Cattolica del Sacro Cuore, 00168 Rome, Italy; 3 Nephrology, Dialysis and Transplantation Unit, Fondazione Policlinico Universitario A. Gemelli IRCCS, 00168 Rome, Italy; 4 Division of Pediatrics, Department of Clinical Science, Intervention and Technology (CLINTEC), Karolinska Institutet, 17177, Stockholm, Sweden

**Keywords:** NSE, BDNF, BBB, Biomarkers, Pediatric CKD

## Abstract

**Background:**

Chronic kidney disease (CKD) in children exposes the developing brain to uremic and vascular insults, yet blood-based markers of the pediatric kidney–brain axis remain underexplored. We characterized neuron-specific enolase (NSE) and brain-derived neurotrophic factor (BDNF) across the spectrum of pediatric kidney disease.

**Methods:**

Prospective cohort of 75 children with measured GFR: 12 comparators with normal kidney function, 31 with non-dialysis CKD stages G2–G5, and 32 kidney transplant recipients (KTx). Biomarkers were measured by ELISA at baseline and after 3.2 years. Cross-sectional and longitudinal analyses adjusted for age and group.

**Results:**

NSE correlated inversely with age (r = − 0.46, *p* < 0.001) and did not differ between groups after age adjustment. NSE–age slopes were comparable in comparators and CKD but flat in transplanted children, indicating loss of the developmental decline in this group. BDNF correlated positively with measured GFR (r = 0.31, p = 0.018) and was lower in CKD than in comparators, consistent with reduced neurotrophic reserve in pediatric uremia.

**Conclusions:**

Circulating NSE in childhood reflects developmental stage rather than CKD status. Group comparisons in pediatric biomarker studies require age adjustment. Transplantation alters the NSE–age relationship beyond what kidney function explains. BDNF tracks kidney function in pediatric CKD. Age-stratified reference intervals are required before either marker can guide clinical decisions.

**Graphical Abstract:**

A higher resolution version of the Graphical abstract is available as Supplementary information

**Figure:**
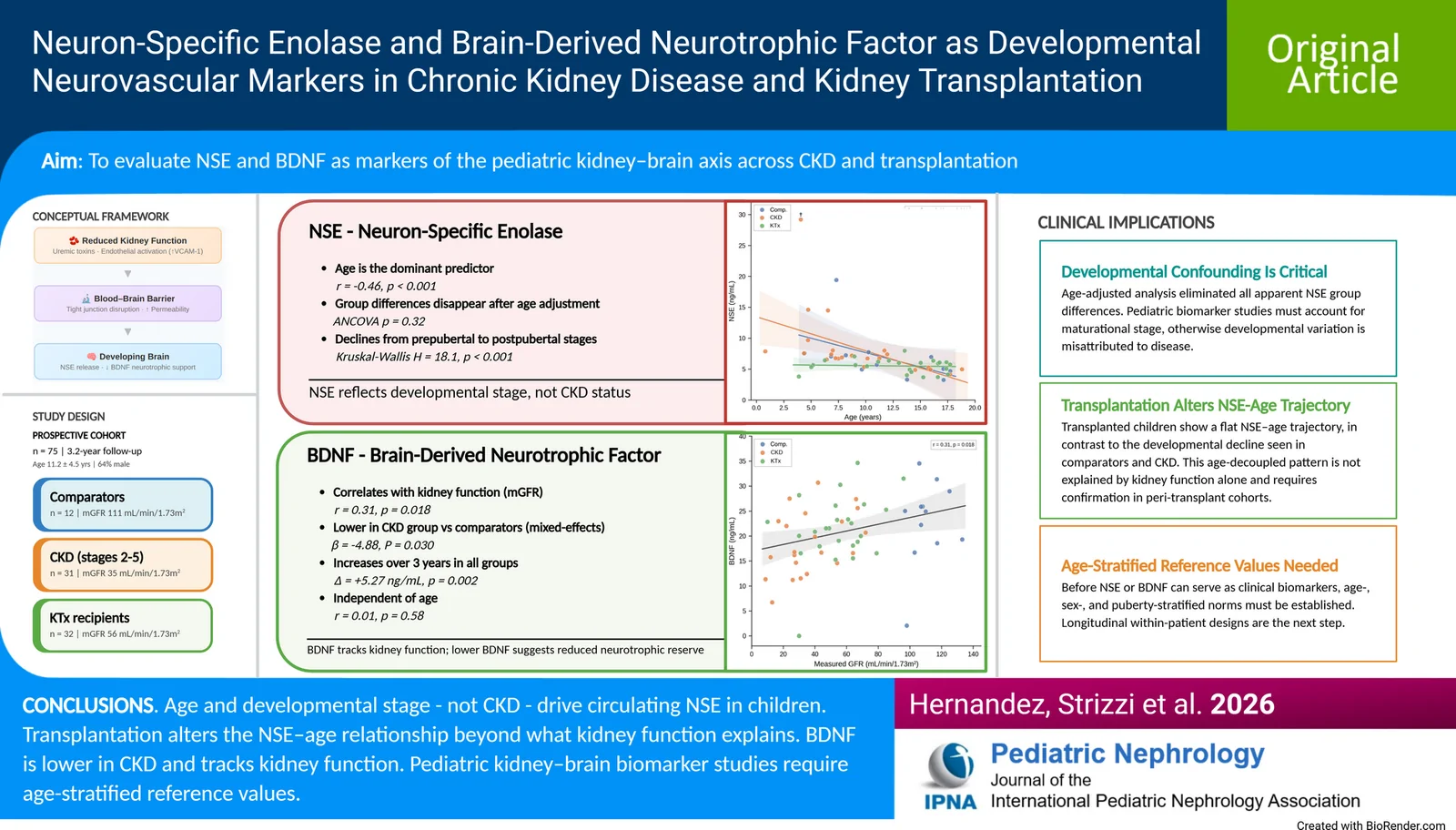

**Supplementary Information:**

The online version contains supplementary material available at https://doi.org/10.1007/s00467-026-07437-w.

## Introduction

Chronic kidney disease (CKD) in children is frequently accompanied by neurological complications that adversely affect neurodevelopment, cognitive function and overall quality of life [1, 2]. In contrast to adult CKD, pediatric CKD exposes the developing brain to chronic kidney-related insults during critical windows of maturation [3, 4].

Early cognitive impairment disproportionately affects attention, executive function, processing speed, and academic performance [5]. Accumulating evidence implicates a kidney–brain axis wherein uremic toxicity, inflammation, and vascular dysfunction converge at the blood–brain barrier (BBB) [6–9]. Endothelial activation and glycocalyx shedding associate with cognitive decline in adult dialysis cohorts and partially reverse after transplantation [10–13].

Current clinical risk stratification relies on estimated GFR, which fails to predict neurovascular complications with sufficient precision. Blood-based biomarkers reflecting neurovascular injury in pediatric CKD remain unexplored, representing a critical gap in kidney–brain axis research.

Neuron-specific enolase (NSE), a neuronal glycolytic enzyme, enters the circulation following neuronal injury or increased BBB permeability [14, 15]; elevated concentrations have been reported in adult hemodialysis cohorts in association with reduced BBB tight-junction protein expression [9]. Brain-derived neurotrophic factor (BDNF), a regulator of synaptic plasticity and neuronal survival, is reduced in adult CKD and dialysis cohorts [9]. BDNF is also expressed in renal cells and detectable in urine, where its mRNA correlates with kidney injury markers [16].

A mechanistic link between endothelial activation and neuronal biomarkers is supported by converging evidence. VCAM-1, elevated in CKD, directly disrupts brain endothelial barrier integrity in experimental models [17] and predicts cognitive decline in adult dialysis cohorts [10, 11]. Kidney function regulates endothelial activation bidirectionally: VCAM-1 rises after nephrectomy and falls after transplantation [12, 13], suggesting a pathway from reduced GFR through endothelial activation to BBB compromise.

Pediatric data are sparse. A single study reported lower serum and higher urinary BDNF in conservatively managed CKD [18]. No study has examined NSE and BDNF across the spectrum of pediatric kidney disease or integrated these markers with endothelial activation and measured kidney function. Without such biomarkers, clinicians lack tools to identify children at highest neurocognitive risk during vulnerable developmental windows.

We aimed to (1) compare NSE and BDNF concentrations across kidney-function-normal comparators, children with CKD, and kidney transplant recipients; (2) identify cross-sectional correlates including kidney function, endothelial activation, and developmental stage; and (3) characterize longitudinal biomarker trajectories over 3 years. We hypothesized that NSE would be elevated in CKD, reflecting neurovascular stress, and would correlate with endothelial activation markers rather than GFR; and that BDNF would track kidney function, reflecting neurotrophic reserve.

## Methods

### Study design and participants

This is a prospective, observational cohort study conducted at the Pediatric Nephrology Unit, Astrid Lindgren Children's Hospital, Karolinska University Hospital Huddinge, Stockholm, Sweden. Seventy-five children were enrolled between 2007 and 2008 in three groups: kidney-function-normal comparators (n = 12), children with CKD stages 2–5 non-dialysis (n = 31), and kidney transplant recipients (KTx, n = 32). Underlying etiologies are presented in Table 1. All KTx recipients were receiving tacrolimus and prednisolone at baseline. Data on additional immunosuppression (mycophenolate mofetil or azathioprine) were not systematically recorded. Comparators were children with normal mGFR seen at the renal outpatient clinic (urinary tract dilatation/VUR 47%, cystic kidney disease 21%, other 32%). Exclusion criteria were ongoing dialysis, congenital or structural myocardial disease, overt heart failure, HIV or hepatitis C infection, or unstable clinical condition. The cohort and recruitment procedures have been described in detail previously [19]. Participants underwent baseline (T0) and follow-up (T1) assessments at a mean interval of 3.2 ± 0.3 years, with measurement of circulating neuronal biomarkers (NSE, BDNF) and clinical parameters. During follow-up, seven patients with CKD underwent kidney transplantation.

**Table 1:** Baseline characteristics of study participants

Variable	Comparators (*n* = 12)	CKD (*n* = 31)	KTx (*n* = 32)	p
**Demographics**				
Age (years)	11.6 ± 4.6	9.9 ± 4.4	13.7 [9.8–16.1]	0.087
Male sex, n (%)	6 (50)	22 (71)	20 (62)	0.454
Pubertal stage, n (%)				0.028
Prepubertal	7 (58)	20 (65)	9 (28)	
Pubertal	1 (8)	6 (19)	13 (41)	
Postpubertal	4 (33)	5 (16)	10 (31)	
**Underlying aetiology, n (%)**				
CAKUT	—	12 (38)	13 (39)	
Glomerulonephritis	—	5 (15)	2 (7)	
Congenital nephrotic syndrome	—	0 (0)	6 (18)	
Vasculitis	—	3 (9)	1 (5)	
FSGS	—	2 (6)	2 (7)	
Juvenile nephronophthisis	—	1 (3)	3 (9)	
Polycystic kidney disease	—	1 (3)	3 (9)	
HUS	—	2 (6)	0 (0)	
Other	—	7 (21)	2 (7)	
**Immunosuppression (KTx)**				
Tacrolimus + prednisolone	—	—	32 (100)	
**Anthropometry**				
Weight Z-score	0.3 ± 0.9	− 0.1 ± 1.1	0.1 ± 1.0	0.482
Height Z-score	− 0.7 ± 1.0	− 0.3 ± 1.1	− 0.6 ± 0.9	0.471
BMI Z-score	0.3 ± 1.0	0.2 ± 1.2	0.6 ± 1.0	0.199
**Kidney function**				
mGFR (mL/min/1.73 m^2^)	110.7 ± 10.7	30.0 [20.0–43.0]	56.1 ± 19.7	< 0.001
eGFR (mL/min/1.73 m^2^)	107.3 ± 12.5	34.6 [22.1–39.7]	55.8 ± 18.3	< 0.001
Cystatin C (mg/L)	0.8 ± 0.1	2.6 ± 1.0	1.6 [1.4–1.9]	< 0.001
Creatinine (µmol/L)	49.5 ± 12.4	156 [111–199]	90.5 [72.5–128]	< 0.001
Urea (mmol/L)	4.3 ± 0.9	18.2 ± 8.7	9.3 [6.7–12.7]	< 0.001
Proteinuria, n (%)	1 (8)	20 (65)	10 (31)	< 0.001
**Haematology and biochemistry**				
Haemoglobin (g/L)	132 ± 12	119 ± 13	123 ± 13	0.012
WBC (× 10⁹/L)	5.3 [5.2–6.3]	6.3 ± 1.8	7.6 [5.9–9.1]	0.005
Albumin (g/L)	40.8 ± 2.6	39.0 [38.0–41.5]	40.2 ± 3.1	0.309
Glucose (mmol/L)	4.8 ± 0.5	4.8 ± 0.6	4.7 [4.5–5.0]	0.909
Calcium (mmol/L)	2.4 ± 0.1	2.4 ± 0.1	2.4 ± 0.1	0.161
Phosphate (mmol/L)	1.4 [1.2–1.5]	1.5 ± 0.3	1.4 [1.2–1.5]	0.183
Homocysteine (µmol/L)	7.5 [6.1–9.5]	14.0 [10.3–18.0]	12.0 [9.3–17.0]	0.001
**Lipid profile**				
Triglycerides (mmol/L)	0.7 ± 0.2	1.0 [0.7–1.6]	1.2 [0.8–1.4]	0.004
Total cholesterol (mmol/L)	4.0 ± 0.7	4.6 ± 1.0	3.9 ± 0.8	0.007
HDL (mmol/L)	1.5 ± 0.4	1.5 ± 0.4	1.4 [1.2–1.6]	0.697
LDL (mmol/L)	2.0 ± 0.5	2.6 ± 0.9	1.9 ± 0.7	0.004
**Cardiovascular**				
SBP (mmHg)	108 ± 11	109 ± 14	113 ± 15	0.492
DBP (mmHg)	63 ± 5	66 ± 10	67 ± 10	0.466
Hypertension, n (%)	0 (0)	7 (24)	9 (28)	0.137
**Mineral metabolism**				
PTH (ng/L)	42.8 ± 18.5	111 [71–162]	75.5 [47.5–88.8]	< 0.001
FGF23 (RU/mL)	58.4 ± 11.9	196 [104–417]	112 [89.5–148]	< 0.001
Klotho (pg/mL)	1818 ± 750	1458 ± 501	1908 ± 950	0.078
**Inflammatory and endothelial markers**				
hsCRP (mg/L)	0.4 [0.2–0.9]	0.4 [0.2–0.9]	0.4 [0.2–2.2]	0.551
VCAM-1 (ng/mL)	777 ± 161	1019 ± 222	879 ± 157	0.002
ICAM-1 (ng/mL)	259 ± 50	244 ± 57	221 ± 76	0.234
**Neuronal biomarkers**				
NSE (ng/mL)	6.3 [4.9–7.3]	6.9 [6.0–7.9]	5.5 ± 1.1	0.007
BDNF (ng/mL)	23.0 ± 8.4	19.3 ± 7.0	21.4 ± 6.7	0.136

Data are presented as mean ± SD for normally distributed variables or median [IQR] for skewed variables. Categorical data are n (%). Normality was assessed by Shapiro–Wilk test. Between-group comparisons: ANOVA or Kruskal–Wallis for continuous variables; χ^2^ or Fisher’s exact test for categorical variables. Comparators: kidney-function-normal children seen at the renal outpatient clinic. mGFR: measured glomerular filtration rate (iohexol or inulin clearance). eGFR: estimated GFR (bedside Schwartz formula)

### Definitions and measurements

#### Glomerular filtration rate

GFR was primarily measured by exogenous filtration markers: iohexol clearance (58%) or inulin clearance (36%). One control used cystatin C-based GFR. eGFR was calculated using the bedside Schwartz formula (eGFR = 0.413 × height (cm)/serum creatinine (mg/dL). KDIGO stages (G1 to G5) were assigned based on mGFR.

#### Anthropometry and pubertal stages

Anthropometric z-scores were calculated. Pubertal stage was assessed by Tanner staging (clinical examination); when unavailable, it was estimated from pubertal hormones, growth charts, and epiphyseal plate radiographs [19].

#### Sample collection and biomarker measurement

Blood samples were collected by venipuncture into serum separator tubes, allowed to clot at room temperature, centrifuged, and aliquoted for storage at − 80 °C at the Karolinska University Hospital Biobank. Per institutional protocol, samples typically underwent one to two freeze–thaw cycles. All measurements were performed within a single analytical campaign (January 2023). NSE and BDNF were quantified by sandwich ELISA (DENL20 and DBD00, respectively; R&D Systems, UK) according to manufacturer protocols. Calibration standards were run in duplicate, patient samples in singlet due to limited serum volume. Intra-assay CVs were 2.9% (NSE) and 4.9% (BDNF). Inter-assay CVs for NSE, from QC samples across plates, ranged from 5.7% to 11.9%; all QC values fell within manufacturer-specified ranges. Hemolyzed samples were excluded by visual inspection.

### Sample size and power

Sample size was determined by feasibility given the single-center design and longitudinal follow-up requirements.

### Statistical analysis

Continuous variables were assessed for normality using Shapiro–Wilk test and visual inspection. Normally distributed data are presented as mean ± SD, non-normal data as median with interquartile range. Between-group comparisons used one-way ANOVA or Kruskal–Wallis test followed by Dunn's post-hoc test for pairwise comparisons with Bonferroni correction; categorical variables were compared using χ^2^ or Fisher's exact test. To account for age differences between groups, analysis of covariance (ANCOVA) was performed for baseline NSE with group as the factor and age as a continuous covariate; age-adjusted estimated marginal means with 95% confidence intervals are reported. Bivariate associations were assessed using Pearson or Spearman correlation as appropriate. Variables with p < 0.20 in univariate analysis were considered for multivariable linear regression models; mGFR was used as the primary kidney function variable in all models. Longitudinal within-subject changes were assessed using paired t-tests or Wilcoxon signed-rank tests. Predictors of the magnitude of change were assessed separately using multivariable linear regression with absolute change (T1 − T0) as the dependent variable and baseline biomarker value, age, sex, and mGFR as covariates. Longitudinal trajectories were additionally analysed using linear mixed-effects models (LMM) with random intercepts for each participant, fitted by restricted maximum likelihood. Fixed effects included time (baseline vs. follow-up), group, age, and sex; time-by-group interaction terms were tested to evaluate differential trajectories. Sensitivity analyses excluded influential observations to assess robustness of key findings. Two-tailed p < 0.05 was considered statistically significant. Given the exploratory nature of this study, we did not adjust for multiple comparisons. Analyses were performed using Python 3.12 (scipy, statsmodels).

### Ethics approval and consent

The study was approved by the Regional Ethical Review Board in Stockholm (2005/977–31/3) and conducted in accordance with the Declaration of Helsinki. Written informed consent was obtained from parents/guardians; children aged ≥ 15 years provided written assent. No participants were ≥ 18 at enrolment.

## Results

### Participant flow

Seventy-five pediatric patients were enrolled: 12 kidney-function-normal comparators, 31 affected by CKD, and 32 kidney transplant recipients (KTx). Mean age at baseline was 11.2 ± 4.5 years (range 0.8–18.8), with 64% male participants. Follow-up occurred 3.2 ± 0.4 years later. Seven patients with CKD underwent transplantation during follow-up. Complete NSE and BDNF data were available for 59 participants at baseline (79%) and 44 at follow-up (59%); 28 participants (37%) had paired longitudinal measurements (missingness due to insufficient sample volume; no significant differences in age, sex, or mGFR between complete and incomplete cases).

### Kidney function

GFR was measured by exogenous filtration markers (iohexol 58%, inulin 36%) in 72/75 patients (96%). Mean mGFR was 111 ± 11 mL/min/1.73 m^2^ in comparators, 35 ± 19 in CKD, and 56 ± 19 in KTx (ANOVA F = 76.4, p < 0.001). KDIGO staging showed all comparators at G1, CKD patients predominantly G3b–G5 (86% with mGFR < 60), and KTx patients at G2–G3a (69%). Cystatin C validated GFR assessment (r = − 0.84, p < 0.001). Bland–Altman analysis showed Schwartz eGFR formula had acceptable bias (− 0.98 mL/min/1.73 m^2^) but wide limits of agreement (± 22 mL/min/1.73 m^2^), misclassifying 26% by KDIGO stage compared to mGFR.

### Baseline neurobiomarker levels

**Fig. 1:**
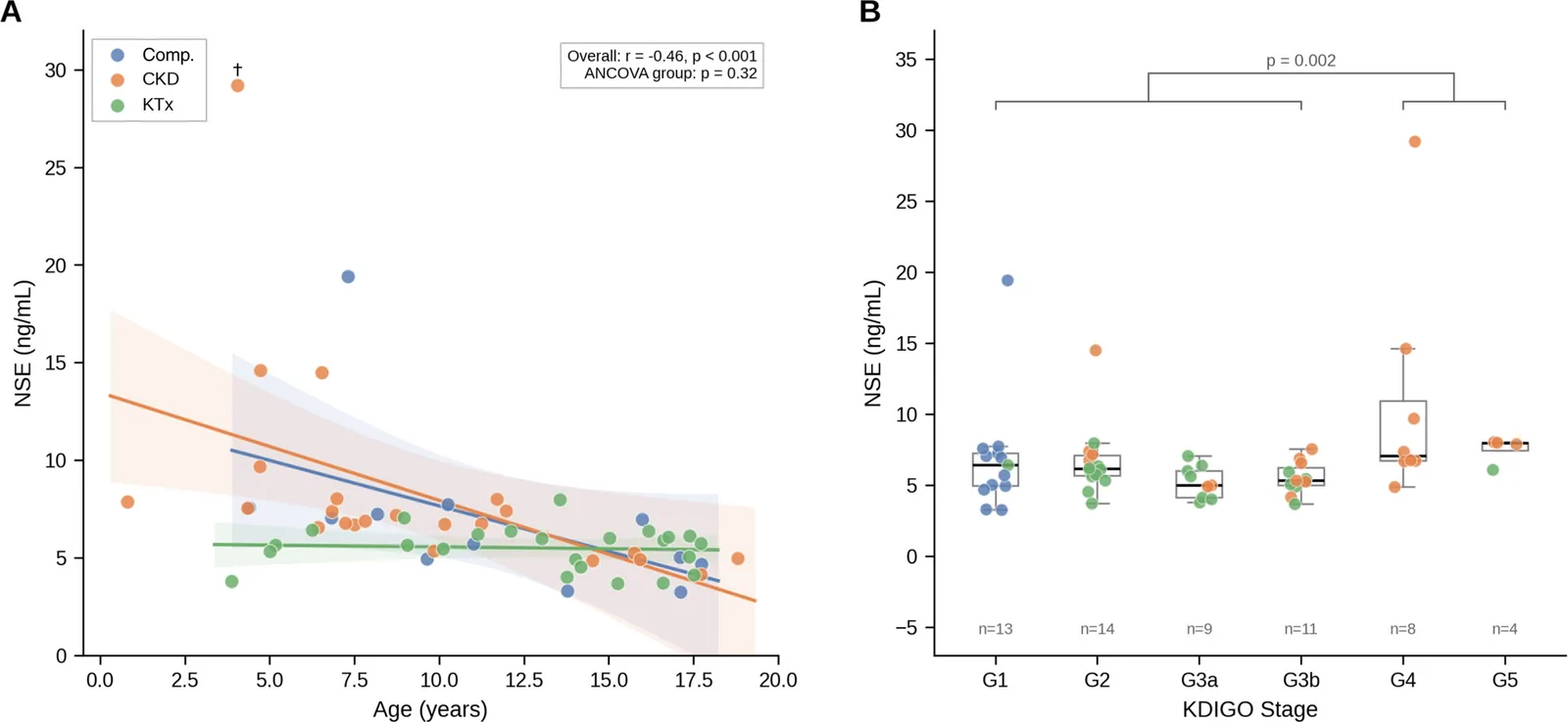
Age as the dominant determinant of NSE in paediatric kidney disease. **A** NSE versus age, stratified by group. Individual data points are colour-coded (Comparator: blue; CKD: orange; KTx: green). Lines represent group-specific linear regression with 95% confidence bands. NSE declined with age across the cohort (overall r = − 0.46, *p* < 0.001); group-specific slopes were concordant between comparators (β = − 0.47 ng/mL/y, 95% CI [− 1.02, + 0.09]) and CKD (β = − 0.55, 95% CI [− 0.99, − 0.11]) but markedly flatter in KTx (β = − 0.02, 95% CI [− 0.13, + 0.09]). Age × group interaction: ANCOVA F(2,53) = 3.01, p = 0.058 (p = 0.032 after sensitivity exclusion of one influential CKD observation, marked with †). **B** NSE by KDIGO stage. Box plots display median and interquartile range; individual data points colour-coded by group. NSE was higher at stages G4–G5 versus G1–G3b (p = 0.002), but children at advanced stages were younger (mean 7.9 vs. 12.1 years), precluding definitive attribution to a GFR threshold independent of age. Sample sizes indicated below each stage

NSE differed across groups in unadjusted analysis (Kruskal–Wallis H = 10.0, p = 0.007): CKD 8.3 ± 5.2 ng/mL, comparators 6.9 ± 4.2, KTx 5.5 ± 1.1. Post-hoc testing identified a significant difference between CKD and KTx (p = 0.001), but not between comparators and CKD (p = 0.26) or comparators and KTx (p = 0.40). The CKD group was younger than the KTx group (9.3 ± 4.7 vs. 12.8 ± 4.4 years) with differing pubertal distributions (p = 0.028), and NSE correlated strongly with age (r = − 0.46, p < 0.001). ANCOVA adjusting for age eliminated the between-group difference (Type II F = 1.15, p = 0.32): age-adjusted means were 7.05 ng/mL (95% CI 4.98–9.12) for comparators, 7.70 (6.15–9.24) for CKD, and 6.04 (4.54–7.54) for KTx. No pairwise contrast reached significance (CKD vs. comparators p = 0.62; CKD vs. KTx p = 0.14; comparators vs. KTx p = 0.43). Age was the sole significant predictor (β = − 0.33, p = 0.002; Fig. 1A). The age × group interaction was non-significant in the full sample (F(2,53) = 3.01, p = 0.058). Sensitivity exclusion of one influential CKD observation (NSE = 29.2 ng/mL in a 4-year-old child with CKD G4) yielded p = 0.032; this is reported as a robustness check and does not alter the primary conclusion of a non-significant interaction. Group-specific slopes were concordant between comparators and CKD (β = − 0.47 and − 0.55 ng/mL/y, respectively) indicating no age-differential NSE excess in CKD across the pediatric range. The KTx slope was markedly flatter (β = − 0.02). The KTx-versus-comparator slope contrast was non-significant in the full sample (p = 0.114) and reached conventional significance only on outlier-excluded sensitivity (p = 0.019) and is therefore reported as hypothesis-generating. NSE was stable across KDIGO stages G1–G3b but elevated at G4–G5 (6.18 ± 2.66 vs. 9.66 ± 6.62 ng/mL; p = 0.002; Fig. 1B); however, children at stages G4–G5 were substantially younger (mean age 7.9 vs. 12.1 years), and with only 8 and 4 patients at G4 and G5 respectively, age confounding cannot be excluded. Sensitivity analysis excluding one CKD participant with markedly elevated NSE (29.2 ng/mL) confirmed these findings (group p = 0.26; age β = − 0.24, p = 0.001).

**Fig. 2:**
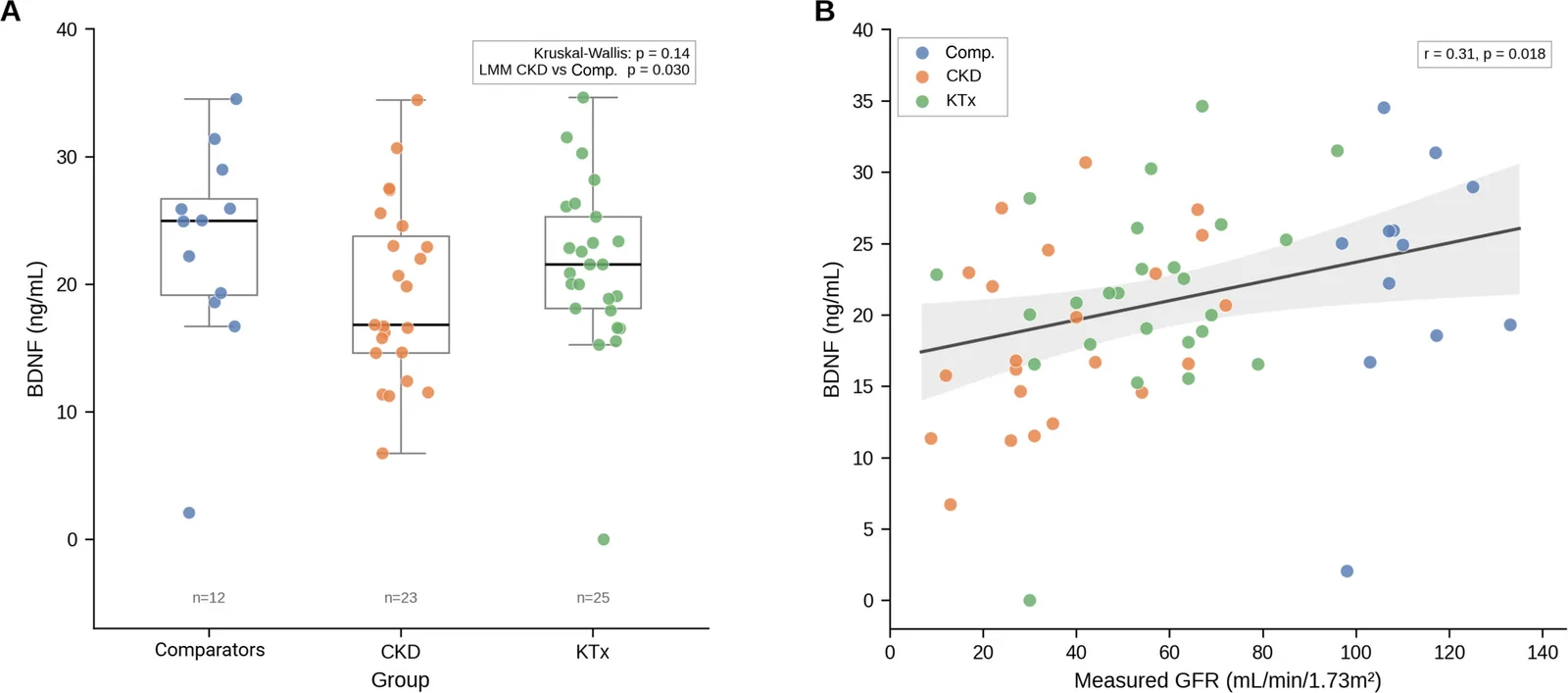
BDNF correlates with kidney function and is lower in CKD. **A** BDNF by group. Box plots display median and interquartile range with individual data points. The cross-sectional Kruskal–Wallis test was not significant (p = 0.14); however, linear mixed-effects modelling leveraging both timepoints identified significantly lower BDNF in CKD compared with comparators (β = − 4.88, p = 0.030). **B** BDNF versus measured GFR (mGFR), stratified by group. Solid line represents overall linear regression with 95% confidence band (r = 0.31, p = 0.018). Data points colour-coded by group (Comparator: blue; CKD: orange; KTx: green). Sample sizes indicated below each group

BDNF did not differ across groups in cross-sectional analysis (Kruskal–Wallis H = 4.0, p = 0.14; Fig. 2A): comparators 23.0 ± 8.4 ng/mL, CKD 19.3 ± 7.0, KTx 21.4 ± 6.7; see Mixed-Effects Models for longitudinal analysis. NSE and BDNF were not correlated at baseline (r = 0.13, p = 0.34; Supplementary Fig. 1).

KTx recipients had unadjusted NSE similar to comparators despite mGFR approximately half that of comparators (56 vs. 111 mL/min/1.73 m^2^); age-adjusted analysis confirmed no KTx–comparator difference (p = 0.43). VCAM-1 showed a graded pattern across groups (comparators < KTx < CKD; H = 10.0, p = 0.007), with CKD significantly higher than comparators (p = 0.005). Seven patients with CKD underwent transplantation during follow-up; paired biomarker data were insufficient for pre-post analysis.

### Correlates of baseline NSE

Univariate correlates of NSE included younger age, prepubertal stage, male sex, elevated VCAM-1, and higher phosphate (Table 2; Supplementary Figs. 1–2). NSE did not correlate with mGFR (r = − 0.10, p = 0.43) or hsCRP (ρ = − 0.14, p = 0.30). Multivariable linear regression (Table 3; n = 50, R^2^ = 0.33, adjusted R^2^ = 0.25, F = 4.3, p = 0.003) identified age (β = − 0.34, SE = 0.13, p = 0.009) and VCAM-1 (β = 0.0078, SE = 0.0031, p = 0.015) as independent predictors. Sex, mGFR, and hemoglobin were not significant (all p > 0.2). Group assignment was not independently associated with NSE when age was included in the model. One child with severe CKD (4-year-old male, mGFR 24 mL/min/1.73 m^2^, KDIGO G4) exhibited markedly elevated NSE (29.2 ng/mL) and VCAM-1 (1430 ng/mL), representing the extreme of the disease spectrum. Sensitivity analysis excluding this participant attenuated the NSE–VCAM-1 correlation to non-significance (r = 0.19, p = 0.19); in the multivariable model, VCAM-1 also lost significance (p = 0.39), while age remained significant (p = 0.003). This association therefore requires validation in larger cohorts with adequate representation of advanced pediatric CKD.

**Table 2:** Univariate correlations with baseline neuronal biomarkers

Biomarker	Variable	Statistic	*p-value*
NSE	Age	r = − 0.46	< *0.001*
NSE	Pubertal stage	H = 18.1	< 0.001
NSE	Male sex	U = 470	0.032
NSE	VCAM-1*	r = 0.43*	0.002
NSE	Phosphate	r = 0.28	0.036
NSE	Measured GFR	r = − 0.10	0.43
BDNF	Measured GFR	r = 0.31	0.018
BDNF	Proteinuria	U = 288	0.030
BDNF	FGF23	ρ = − 0.37	0.005

*NSE* neuron-specific enolase, *BDNF* brain-derived neurotrophic factor

*r* Pearson correlation, *H* Kruskal–Wallis statistic, *U* Mann–Whitney U, *ρ* Spearman correlation

^*^The NSE–VCAM-1 association was substantially influenced by one participant with severe CKD and concordant biomarker elevations; sensitivity analysis excluding this case attenuated the correlation to non-significance (r = 0.19, p = 0.19). See Discussion for detailed interpretation

**Table 3:** Multivariable linear regression analysis: predictors of neuronal biomarkers

Variable	β	SE	95% CI	p	
**NSE (baseline)**					
Age (years)	− 0.3447	0.1256	[− 0.598, − 0.092]	0.009	**
Sex (male)	− 0.0150	1.2779	[− 2.591, 2.561]	0.991	
mGFR (mL/min/1.73 m^2^)	0.0039	0.0194	[− 0.035, 0.043]	0.843	
Haemoglobin (g/L)	0.0634	0.0504	[− 0.038, 0.165]	0.216	
VCAM-1 (ng/mL)°	0.0078	0.0031	[0.002, 0.014]	0.015	*
*Model statistics*	*N* = *50 R*^*2*^ = *0.327 Adj R*^*2*^ = *0.251*				
**BDNF (baseline)**					
Age (years)	0.0349	0.2034	[− 0.373, 0.442]	0.864	
Sex (male)	− 0.2393	2.1753	[− 4.597, 4.118]	0.913	
mGFR (mL/min/1.73 m^2^)	0.0661	0.0287	[0.009, 0.124]	0.025	*
*Model statistics*	*N* = *60 R*^*2*^ = *0.094 Adj R*^*2*^ = *0.045*				
**ΔNSE**					
Baseline NSE (ng/mL)	− 1.0626	0.1695	[− 1.413, − 0.712]	< 0.001	***
Age (years)	− 0.3421	0.2025	[− 0.761, 0.077]	0.105	
Sex (male)	− 3.3965	1.7204	[− 6.955, 0.162]	0.061	
mGFR (mL/min/1.73 m^2^)	− 0.0192	0.0232	[− 0.067, 0.029]	0.417	
*Model statistics*	*N* = *28 R*^*2*^ = *0.661 Adj R*^*2*^ = *0.602*				
**ΔBDNF**					
Baseline BDNF (ng/mL)	− 0.7112	0.1560	[− 1.033, − 0.389]	< 0.001	***
Age (years)	0.0635	0.3090	[− 0.574, 0.701]	0.839	
Sex (male)	− 6.2709	2.8898	[− 12.235, − 0.307]	0.040	*
mGFR (mL/min/1.73 m^2^)	0.0200	0.0405	[− 0.064, 0.104]	0.627	
*Model statistics*	*N* = *29 R*^*2*^ = *0.532 Adj R*^*2*^ = *0.454*				

Significance levels: * p < 0.05, ** p < 0.01, *** p < *0.001*

*β* unstandardised regression coefficient, *SE* standard error, *CI* confidence interval

All baseline models adjusted for age and sex. Longitudinal models additionally adjusted for respective baseline biomarker value. mGFR was used as the primary kidney function variable in all models

°The NSE–VCAM-1 association in the multivariable model was substantially influenced by one participant with severe CKD and concordant biomarker elevations; leave-one-out sensitivity analysis excluding this case attenuated the association to non-significance (β = 0.0019, SE = 0.0022, p = 0.39), while age remained an independent predictor (p = 0.003). Given the dependence on a single observation, the NSE–VCAM-1 association should be regarded as exploratory and hypothesis-generating rather than as evidence of an independent predictor; we have retained it in the table to preserve transparency of the full model rather than to assert predictor status. See Discussion for interpretation

### Correlates of baseline BDNF

Univariate correlations (Table 2) showed BDNF associated with mGFR (r = 0.31, p = 0.018; Fig. 2B), FGF23 (ρ = − 0.37, p = 0.005), and proteinuria (Mann–Whitney p = 0.030). Unlike NSE, BDNF did not correlate with age (r = 0.07, p = 0.58; Supplementary Fig. 1) or inflammatory markers (hsCRP ρ = 0.00, p = 0.98). FGF23 correlated strongly with mGFR (ρ = − 0.85, *p* < 0.001).

Multivariable linear regression including age, sex, and mGFR (Table 3; n = 60, R^2^ = 0.09, adjusted R^2^ = 0.05, F = 1.9, p = 0.13) showed mGFR as the only nominally significant predictor (β = 0.066, SE = 0.029, p = 0.025), though the model was not globally significant, reflecting the limited explanatory power of available covariates for BDNF variation. FGF23 did not contribute independently when added (p = 0.88), consistent with GFR mediation.

### Longitudinal changes in neuronal biomarkers

**Fig. 3:**
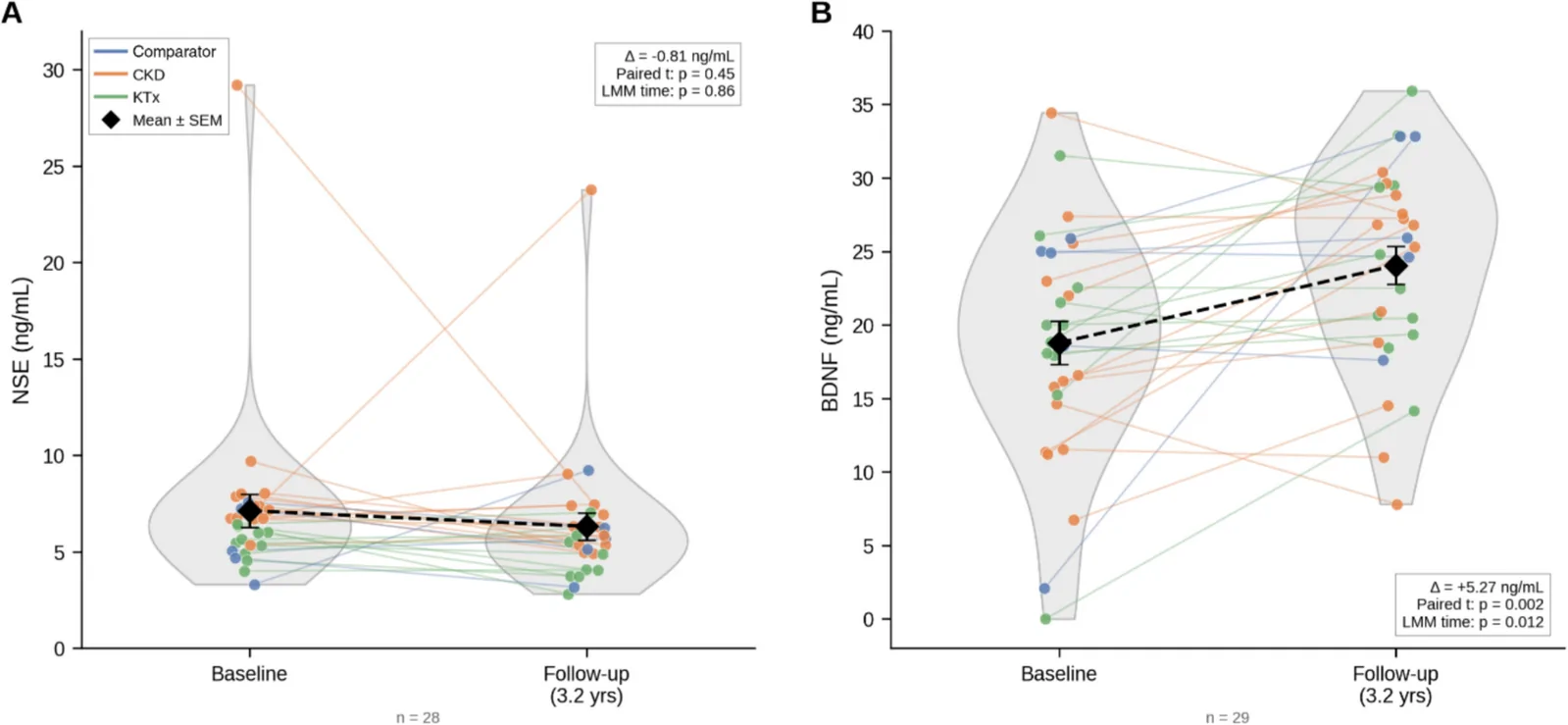
Longitudinal biomarker trajectories over 3.2 years. **A** NSE remained stable (Δ = − 0.81 ng/mL, paired t p = 0.45; LMM time effect p = 0.86). **B** BDNF increased significantly (Δ = + 5.27 ng/mL, paired t p = 0.002; LMM time effect p = 0.012). Violin plots display the distribution at each timepoint. Thin lines connect individual paired measurements, color-coded by group (Comparator: blue; CKD: orange; KTx: green). Black diamonds with error bars indicate overall mean ± SEM; dashed line connects means. Paired analyses: *n* = 28 (NSE) and *n* = 29 (BDNF); LMM results utilize all available observations (*N* = 75 participants)

NSE remained stable over 3.2 ± 0.3 years (7.1 ± 4.5 to 6.3 ± 3.8 ng/mL; Δ − 0.81 ± 5.67; paired t = 0.76, p = 0.45; Fig. 3A). BDNF increased significantly (18.8 ± 7.9 to 24.1 ± 7.0 ng/mL; Δ + 5.27 ± 8.32; t = − 3.41, p = 0.002; Fig. 3B). Percent changes did not differ across groups for either marker (ΔNSE% p = 0.87; ΔBDNF% p = 0.50). Within-subject absolute changes in NSE and BDNF were positively correlated (r = 0.42, p = 0.025); this was attenuated when expressed as percent change (ρ = 0.38, p = 0.054) and after adjustment for respective baseline values (partial r = 0.30, p = 0.13).

### Mixed-effects models

LMMs with random intercepts used all available observations (NSE: 103; BDNF: 104 from 75 participants). For NSE, the LMM confirmed no significant change over time (β = − 0.13, p = 0.86) and no group effects after adjustment for age and sex (CKD vs. comparators p = 0.57; KTx vs. comparators p = 0.29); age remained the sole significant predictor (β = − 0.27, p = 0.003). The longitudinal LMM yielded the same pattern: non-significant interaction (LRT χ^2^(2) = 5.13, p = 0.077) driven by KTx slope flatness.

For BDNF, the LMM confirmed a significant increase over time (β = + 3.41, p = 0.012) with no differential trajectory across groups (time × group interactions all p > 0.49). Notably, the LMM identified lower BDNF in CKD compared with comparators (β = − 4.88, p = 0.030; Fig. 2A), an effect not detected in the cross-sectional analysis (Kruskal–Wallis p = 0.14). The LMM also identified lower BDNF in CKD compared with comparators (β = − 4.88, p = 0.030; Fig. 2A), an effect not detected in the cross-sectional analysis (Kruskal–Wallis p = 0.14). This is a single within-model contrast, uncorrected for multiplicity, at threshold significance in a small cohort, and should be interpreted as hypothesis-generating.

### Predictors of change

For ΔNSE (n = 28, R^2^ = 0.66, adjusted R^2^ = 0.60; Table 3), baseline NSE was the dominant predictor (β = − 1.06, SE = 0.17, p < 0.001), with age (β = − 0.34, SE = 0.20, p = 0.10) and male sex (β = − 3.40, SE = 1.72, p = 0.061) showing trends that did not reach significance. mGFR did not predict ΔNSE (p = 0.42). For ΔBDNF (n = 29, R^2^ = 0.53, adjusted R^2^ = 0.45; Table 3), baseline BDNF predicted change (β = − 0.71, SE = 0.16, p < 0.001), with male sex associated with smaller increases (β = − 6.27, SE = 2.89, p = 0.040). Age and mGFR were not significant (both p > 0.6).

### Other clinical parameters

Anthropometrics (weight Z 0.02 ± 1.05, height Z − 0.50 ± 0.98, BMI Z 0.39 ± 1.09) did not differ across groups (all p > 0.13). Weight Z-score correlated with mGFR (r = 0.23, p = 0.044) but not neurobiomarkers. Hypertension prevalence was 0% in comparators, 24% in CKD, 28% in KTx. Neither NSE nor BDNF differed by hypertension status (p = 0.30 for both). Univariate correlations between systolic BP and NSE (ρ = − 0.29, p = 0.029) and pulse pressure and NSE (ρ = − 0.35, p = 0.008) were attenuated to non-significance after adjustment for age, sex, and disease group. BDNF showed no correlation with any BP parameter (all p > 0.15). Triglycerides correlated inversely with mGFR (r = − 0.39, p < 0.001). hsCRP (1.64 ± 3.39 mg/L) showed no group differences (p = 0.55) or neurobiomarker associations.

## Discussion

### Principal findings

The central finding is that age and pubertal stage, rather than CKD status or kidney function, are the dominant determinants of circulating NSE in children. Unadjusted group comparisons suggested elevated NSE in CKD, but this difference was entirely attributable to the younger age composition of the CKD group and was eliminated after adjustment. BDNF correlated with mGFR, and the LMM identified lower BDNF in CKD compared with comparators (p = 0.030), an effect not detected in the underpowered cross-sectional analysis. Whether early-onset CKD constitutes a distinct neurodevelopmental risk window remains an open question, best addressed in larger studies incorporating tests with cognitive outcomes.

### NSE as a marker of endothelial activation

Age-adjusted analysis eliminated the between-group NSE difference (p = 0.32), and the absence of an NSE–mGFR correlation is similarly explained by confounding: younger children had both higher NSE and lower mGFR. Group-specific NSE–age slopes were concordant between comparators and CKD across the pediatric range, indicating that CKD does not impose a detectable age-dependent excess; by contrast, the KTx trajectory was essentially flat. NSE enters the circulation following neuronal injury or increased BBB permeability [14, 15]; a disease-related contribution remains biologically plausible but could not be isolated from developmental effects.

The NSE–VCAM-1 association, though significant in the full sample, was not robust to exclusion of a single influential observation and cannot be considered established. We present it as a biologically plausible hypothesis warranting testing in larger cohorts with adequate representation of KDIGO G4–G5 pediatric CKD, a population underrepresented in biomarker research. Of note, VCAM-1 itself correlated inversely with mGFR (r = − 0.44, *p* < 0.001), supporting an indirect pathway (reduced GFR → uremic milieu → endothelial activation → BBB disruption → NSE release).

### Developmental regulation of NSE

Population-based reference intervals confirm higher NSE in younger children [20], consistent with increased NSE expression during neuronal maturation [21]. The age-related NSE decline contrasts with neurofilament light chain (NfL), which increases with age in pediatric CKD and correlates inversely with kidney function and cerebellar grey matter volume [22]. This opposite trajectory suggests complementary processes: NfL may reflect cumulative axonal injury and senescence [23, 24], whereas NSE, a glycolytic enzyme, may capture active neuronal metabolic stress during periods of high cerebral energy demand in childhood [25–27]. Sex-specific reference intervals are also needed, given higher NSE in males in both our data and adult cohorts [28, 29].

These developmental effects have direct methodological consequences: cross-sectional comparisons between groups of differing age composition risk attributing maturational variation to disease. Future pediatric NSE studies require age-, sex-, and puberty-stratified reference values before clinical interpretation is possible.

### Transplantation and NSE normalization

KTx recipients had NSE levels comparable to comparators at any given age, but did not show the developmental decline observed in comparators and CKD. This flatter NSE–age pattern in transplanted children was not significant in the primary full-sample interaction analysis and remains exploratory; nonetheless, an age-decoupled neuronal stress profile after transplantation is biologically coherent with the VCAM-1 reduction and glycocalyx improvement described in adult kidney transplant cohorts. Whether transplantation reshapes the neurovascular interface in ways that uncouple circulating NSE from neurodevelopmental dynamics requires confirmation in longitudinal peri-transplant data.

### BDNF, kidney function and longitudinal dynamism

The LMM suggested that BDNF may be lower in CKD than in comparators (β = − 4.88 ng/mL), an effect not seen in the cross-sectional analysis. This pattern is consistent with the positive BDNF–mGFR association, but rests on a single within-model contrast in a small cohort and requires replication. BDNF is expressed in renal tubules with TrkB receptors in proximal, distal, and collecting duct epithelium [16, 30–32]; circulating BDNF reflects both CNS and peripheral sources, particularly platelets [33, 34]. The association with proteinuria (p = 0.030) raises the possibility that urinary BDNF loss contributes to lower circulating concentrations in CKD.

The uniform BDNF increase over 3 years across all groups, including comparators, is most parsimoniously explained by adolescent neurodevelopment (synaptic pruning, myelination) [35, 36], and argues that single-timepoint BDNF measurements are uninterpretable without age-normed reference values. Disease-related contributions cannot be excluded [9, 18, 37, 38], but are not required to explain the pattern.

### NSE–BDNF temporal associations

NSE and BDNF were uncorrelated at baseline yet within-subject changes were positively correlated (r = 0.42, p = 0.025). This association persisted after adjustment for baseline age (partial r = 0.40, p = 0.034) but attenuated to non-significance after adjustment for baseline biomarker values (r = 0.30, p = 0.13), suggesting that regression to the mean contributes substantially. A shared upstream mechanism, such as BBB permeability fluctuations, given that NSE leaks during barrier disruption [14, 15] and BDNF crosses the BBB bidirectionally [39, 40], remains plausible but unconfirmed. These markers may be better suited to longitudinal monitoring than single-timepoint interpretation.

### Limitations

The sample size (N = 75, with 28–29 paired measurements) restricts power for subgroup analyses; the large number of comparisons without correction increases the risk of type I and type II error, and findings should be interpreted as hypothesis-generating. Comparators were clinic-referred rather than community-recruited, and comparator participation (12/28, 43%) was lower than in CKD and KTx, introducing potential selection bias of undetermined direction; formal exclusion of comparators with cystic kidney disease (two to three subjects) was precluded by subgroup size. Per-subject etiology coding was unavailable; CAKUT and non-CAKUT subgroups (approximately 12 and 19 in CKD, respectively) were too small for stratified modelling, leaving residual phenotypic heterogeneity unaddressed. Samples were stored for 12–16 years at − 80 °C and assayed in singlet owing to volume constraints; per institutional biobank protocol, samples typically underwent one to two freeze–thaw cycles, though the exact count per aliquot was not recorded. Inter-assay CVs for NSE (5.7–11.9%) approached the upper bound for singlet ELISA; within-subject ΔNSE magnitudes approached analytical variability, so individual trajectories require cautious interpretation. The absence of neurocognitive assessments and neuroimaging prevents linkage of biomarker levels to functional brain outcomes, and detailed immunosuppression characterization (mycophenolate, azathioprine) and concomitant CNS diagnoses were not systematically recorded. The single Scandinavian center limits generalizability.

### Clinical implications and future directions

Two categories of clinical consideration emerge, though all require validation. First, regarding what clinicians should consider based on this work. GFR stage alone may be an insufficient proxy for neurocognitive risk: NSE variation was driven by age and developmental stage rather than kidney function, and CKD did not impose a detectable age-dependent excess at the cohort level. Second, what this work suggests for future investigation. Several findings warrant validation as Tier 2 hypotheses: whether the age-decoupled NSE trajectory in KTx reflects a transplant-specific signature; whether lower BDNF in CKD (LMM β = − 4.88, p = 0.030, single contrast uncorrected for multiplicity) reflects compromised neurotrophic reserve; and whether early-onset CKD constitutes a distinct neurodevelopmental risk window when paired with cognitive outcomes. Beyond these, age-, sex-, and puberty-stratified NSE reference values are a prerequisite for clinical use. Longitudinal within-patient designs spanning the peri-transplant period are needed to disentangle transplant effects from maturational trajectories. Future studies should incorporate neurocognitive assessments and neuroimaging as co-primary endpoints to determine whether biomarker deviations from age-expected trajectories predict functional outcomes. Biomarker-guided risk stratification and transplant timing trials, powered for cognitive endpoints, represent the next translational step.

## Conclusions

Three findings emerge for the interpretation of circulating NSE and BDNF in pediatric kidney disease. First, NSE is governed primarily by age and developmental stage: comparators and CKD share a common age-dependent decline, and CKD imposes no detectable age-adjusted excess. Second, transplanted children display a flat NSE–age trajectory, a hypothesis-generating observation that may reflect transplant-associated neurovascular remodelling and requires confirmation. Third, BDNF correlates with kidney function and is lower in CKD, suggesting reduced neurotrophic reserve as a working hypothesis pending replication. Cross-sectional comparisons that ignore developmental stage risk misattributing maturational variation to disease; age-stratified reference values are a prerequisite for biomarker-guided risk stratification in pediatric CKD.

## Supplementary Information

Below is the link to the electronic supplementary material.

Supplementary file1 (DOCX 536 KB)

Graphical abstract (PNG 2757 KB)

## Data Availability

The data supporting the findings of this study are available from the corresponding authors upon reasonable request.
