# Supplementary material for: Neuron-specific enolase and brain-derived neurotrophic factor as developmental neurovascular markers in chronic kidney disease and kidney transplantation
Source: Pediatr Nephrol. 2026 Jul 1;41(11):4199–210. doi: 10.1007/s00467-026-07437-w (PMC13612604; doi:10.1007/s00467-026-07437-w)
Supplement: Supplementary file 1 [file 467_2026_7437_MOESM1_ESM.docx]

**Supplementary Figure 1. Cross-sectional correlations between neuronal biomarkers and clinical variables.** (A) BDNF versus age (r = 0.07, p = 0.58). (B) NSE versus measured GFR (r = −0.10, p = 0.43). (C) NSE versus VCAM-1 (r = 0.43, p = 0.002); sensitivity analysis excluding one outlier attenuated the correlation to non-significance (r = 0.19, p = 0.19). (D) NSE versus BDNF at baseline (r = 0.13, p = 0.34). Data points colour-coded by group (Comparator: blue; CKD: orange; KTx: green). Solid lines indicate significant correlations (p < 0.05); dashed lines indicate non-significance. Shaded areas represent 95% confidence bands.

**Supplementary Figure 2. Effects of pubertal stage and sex on neuronal biomarkers.** (A) NSE by pubertal stage; prepubertal children exhibited significantly higher levels (Kruskal–Wallis p < 0.001). (B) BDNF did not differ by pubertal stage (p = 0.24). (C) Males exhibited higher NSE than females (Mann–Whitney p = 0.032). (D) BDNF did not differ by sex (p = 0.41). Box plots display median and interquartile range. Individual data points are shown with jitter. Sample sizes indicated below each group.
